# Research on the relationship between architectural features in northeast China and vertical aerosol transmission of COVID-19

**DOI:** 10.3389/fpubh.2022.1052610

**Published:** 2023-01-12

**Authors:** Xia Li, Bingxin Sun, Keyang Lyu, Jiayu Chen, Yunjian Zhang, Yu Sun, Chenguang Li, Tianzhuo Sui, Xinxin Wang, Yu Hu, Qin Wang, Dongqun Xu

**Affiliations:** ^1^China CDC Key Laboratory of Environment and Population Health, National Institute of Environmental Health, Chinese Center for Disease Control and Prevention, Beijing, China; ^2^Changchun Center for Disease Control and Prevention, Changchun, Jilin, China; ^3^National Institute of Environmental Health, Chinese Center for Disease Control and Prevention, Beijing, China; ^4^Shandong Provincial Third Hospital, Jinan, Shandong, China

**Keywords:** COVID-19, vertical aerosol transmission, toilet flush, sewage pipe, floor drain, range hood, negative pressure

## Abstract

During the COVID-19 pandemic, many buildings in northeast China have had clusters of infected cases in the vertical layout. There is speculation that vertical aerosol transmission occurs. The houses in northeast China are airtight, and range hoods may be used for a long period of time when cooking. The pathway and factors influencing vertical aerosol transmission are worth studying. To elucidate a viral aerosol transmission pathway, we selected a multistory apartment and a high-rise building in Changchun city, Jilin province, China, to conduct an in-depth investigation and on-site simulation experiments. According to epidemiological investigation information on infected cases, building structures, drainage, ventilation, etc., we used fluorescent microspheres to simulate the behaviors of infected people, such as breathing and flushing the toilet after defecation, to discharge simulated viruses and track and monitor them. The field simulation experiment confirmed the transmission of fluorescent microsphere aerosols to other rooms in two types of buildings using a vertical aerosol transmission pathway of toilet flush-sewage pipe-floor drain without a water seal. Our study showed that, in the absence of a *U*-shaped trap or floor drain water seal whether in a multistory apartment or high-rise residential building, there is a transmission pathway of “excretion of virus through feces-toilet flushing-sewage pipe-floor drain without water seal,” which will cause the vertical transmission of viral aerosol across floors during the COVID-19 pandemic. Moreover, the negative pressure generated by turning on the range hood when closing doors and windows increase aerosol transmission. Based on this negative pressure, prevention and control measures for residential buildings in northeast China during the COVID-19 pandemic were proposed.

## 1. Introduction

Since the COVID-19 outbreak, researchers found many cases of SARS-CoV-2 aerosol transmission in buildings around the world. In a study of infection in three households of the same type on different floors in Guangzhou (1), researchers used tracer gas in the bathroom drainage system to conduct a simulation experiment. The detected tracer gas matched the location where infected people and the nucleic acid-positive environmental samples were found. In an apartment complex in Seoul, South Korea (2), five people with SARS-CoV-2 confirmed infection from three apartments in the same vertical layout reported no direct contact with other residents. Detection and phylogenetic analysis of environmental samples revealed that SARS-CoV-2 transmission might occur between different floors within the same apartment building. Researchers used smoke to generate visual airflow and observed smoke around bathroom drains and vents, which demonstrated the spread of aerosols through non-functional drain traps. Neither tracer gas nor smoke can well-simulate the distribution and aerodynamic characteristics of viral aerosols. Our study group used fluorescent microspheres with different particle sizes to simulate the size range of virus particles discharged from infected people and established a field simulation experimental method for viral aerosol transmission. The method was successfully applied to the field simulation of aerosol transmission in a quarantined hotel in Guangzhou (3) and a high-rise residential building in Shenzhen, China (4). Simulation experiments in a quarantined hotel showed that viral aerosols released by flushing the toilet after defecation and urination not only contaminated rooms on different floors through the bathroom exhaust system and vertical drainage pipe, resulting in cross-layer vertical transmission, but also intensified the transmission to different rooms on the same floor by allowing air from the contaminated room to enter a corridor and then to be sent to different rooms *via* mixing with fresh air. The field simulation experiment conducted in Shenzhen confirmed the existence of a vertical aerosol transmission pathway from toilet flushing-soil stack-floor drains without a water seal. As evidenced by an increase in cases, architectural features of buildings, ventilation in bathrooms, and water seals in floor drains had an important influence on the aerosol transmission of SARS-CoV-2.

Since March 2022, the COVID-19 outbreaks clustered in many residential areas in Changchun, Jilin province, located in northeast China. A common feature of these clusters was that people who were infected lived on different floors of the same vertical layout, while those who lived on the same floor were less infected. Based on a comprehensive analysis of the epidemiological investigation results, nucleic acid testing of environmental samples, and gene sequencing, the possibility of contact transmission was ruled out. Due to the cold winter in northeast China, air tightness of building doors and windows was well-maintained. In winter and early spring, local residents seldom open doors or windows for ventilation and tend to stay indoors for a long time. In high-rise buildings, toilets are frequently used during peak hours. Investigation showed that there were no *U*-shaped traps or water seals in room floor drains. The cooking methods of local residents were mainly stewing, boiling, and frying. Due to long cooking time and increased cooking fumes, kitchen hoods are usually turned on while cooking, which accelerates the flow of air into the exhaust duct.

Vertical aerosol transmission and related influencing factors are worth studying. Therefore, we selected a multistory apartment building and a high-rise residential building to perform simulation experiments using the field simulation experiment methods established by our study group.

## 2. Materials and methods

### 2.1. On-site investigation and identification of research sites

**Figure 1:**
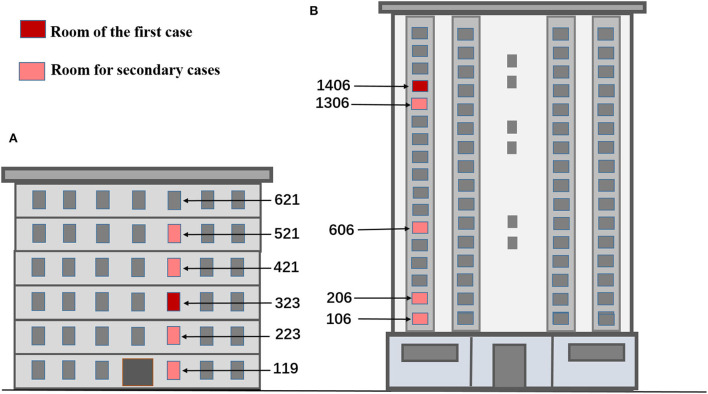
Location map of rooms for simulation experiments based on infection time in buildings **(A, B)**. **(A)** The multistory apartment. **(B)** The high-rise residential building.

Research sites were identified using local epidemiological investigation data, nucleic acid test results, and gene sequencing of cases. According to the epidemiological investigation data and on-site investigation, infected cases in a multistory apartment (building A) were found to be in rooms 119, 223, 323, 421, and 521, which had the same vertical type (no one lived in room 621). The drainage and exhaust systems in these rooms are connected by the same sewage and exhaust standpipe. The first infected case lived in room 323; the result of a self-tested antigen was positive on March 27, 2022, and was transferred to a designated hospital on March 30. During the testing after 2 April, the residents in rooms 119, 223, 521, and 421 were successively diagnosed (Figure 1A). The gene sequencing results of the cases in building A were highly homologous, suggesting the same source of infection.

In a high-rise residential building (building B), the cases who were infected were distributed in rooms 106, 206, 606, 1306, and 1406, which were located in the same vertical layout and connected by the same standpipes of sewage, exhaust and fume, respectively. The first two cases lived in room 1406. The two cases were transferred to a designated hospital as their nucleic acid tests were positive on April 2. Then, the cases who lived in rooms 106, 206, 606, and 1306 were also transferred to a designated hospital as their nucleic acid tests came back positive on 6 April (Figure 1B). Gene sequencing of the cases who were infected in building B was also highly homologous. When the epidemic broke out in the two buildings, all residents followed the home quarantine policy and never went out. All daily supplies and garbage were delivered by special personnel. As the epidemiological investigation found no contact between residents during the outbreak, such as visiting, talking, or sharing an elevator, the possibility of getting the infection from close contacts could be ruled out.

#### 2.1.2. Determination of simulation experimental scenarios

According to the timing of positive nucleic acid screening results and the characteristics of the building structure, drainage, and ventilation, two simulation experimental scenarios were designed, respectively, for buildings A and B. One was exhaling virus simulants (Scenario I), and the other was exhaling while flushing the toilet after defecation to emit viral simulants (Scenario II). In these simulation experiments, fluorescent polystyrene particles (with sizes including 0.3, 0.6, 2, 3, and 6 μm, provided by the Beijing Institute of Metrology) were used as viral simulants as they had aerodynamic properties similar to the SARS-CoV-2 spike pseudovirus. Relevant experimental results have been described in our published paper (5, 6). An aerosol generator of the 6 Jet Collision Nebulizers (BGI, INC) was used to simulate the virus expelled from an infected person. The size of aerosol particles produced using this device ranges from several nanometers to 30 μm (7, 8). These aerosols were generated at a flow rate of 12 L/min, and the exhaled volume of fluorescent particles was 10^12^-10^13^ per hour (9, 10). We used a mixture of different-sized fluorescent polystyrene particles to simulate aerosol clouds expelled during physiological behaviors, such as breathing and sneezing, in line with the real situation in which positive cases expelled viral aerosols. By using an aerosol generator, simulants of different particle sizes can exist independently. To simulate defecation, fluorescent polystyrene particles were poured into the toilet. We revealed a vertical aerosol transmission pathway inside buildings A and B by tracking viral simulants.

#### 2.1.3. Field experimental methods

The two experimental scenarios of building A were as follows. In Scenario I, before the field simulation experiment, swab samples were smeared from toilet surfaces, exhaust air ducts, and floor drains, and differences in temperature, humidity, and air velocity/pressure in air ducts were measured as background values. At the beginning of the experiment, simulants (fluorescent particles) released by an aerosol generator were used to simulate viral exhalation in the bathroom of room 323; the differences in air velocity/pressure in bathroom exhaust air ducts and the number of particles were measured in the bathrooms of rooms 119, 223, 421, 521, and 621 every 10 min. After a 1-h experiment, swab samples were smeared from the toilet surface and the exhaust duct. In Scenario II, simulants were used to simulate viral exhalation and flushing the toilet after defecation in room 323. As there were usually three to eight people living in each room, the toilet was used very frequently during the morning rush hour. In a 1-h experiment in Scenario II, flushing the toilet was also carried out in rooms 119, 223, 421, or 521 as different flushing permutations (no flushing in room 621 because nobody lived there) every 10 min, while the air velocity/pressure differences in bathroom exhaust air ducts and the number of aerosol particles were simultaneously measured. PM_10_ aerosol filter membrane samples were collected for 1 h using medium flow samplers in the bathrooms of rooms 119, 223, 421, and 521. After a 1-h experiment, swab samples were smeared from toilet surfaces, exhaust air ducts, and floor drains (see Supplementary Table 1 for details).

We also conducted two experimental scenarios in building B (due to site conditions, rooms 206, 806, 1206, 1406, 1606, and 1706 were selected for the experiment). In Scenario I, before the field simulation experiment, swab samples were smeared from toilet surfaces, exhaust fans, and floor drains, and the differences in temperature, humidity, air velocity/pressure between floor drains, and exhaust fans were measured as background values. In the bathroom of room 1206, simulants (fluorescent particles) released by an aerosol generator were used to simulate exhaled viruses, while the bathroom door and kitchen range hoods and exhaust fans were all closed. Most families in the building have three meals a day at the same time, especially when staying at home due to the epidemic. In northeast China, people tend to stew, boil, or fry a variety of foods, which would require more cooking time, and range hoods in the kitchen need to be turned on when cooking. Therefore, during the experiment, all kitchen range hoods and bathroom doors of rooms 206, 806, 1406, 1606, and 1706 were opened (doors of a separate kitchen were opened) to simulate the scenario. The differences in air velocity/pressure in the exhaust fans and floor drains as well as the number of aerosol particles in the bathrooms of rooms 206, 806, 1406, 1606, and 1706 were measured every 10 min. PM_10_ aerosol filter membrane samples were also collected for 1 h with medium flow samplers in bathrooms. After a 1-h experiment, swab samples were smeared from toilet surfaces, exhaust fans, and floor drains. In Scenario II, simulants were used to simulate exhaled viruses and toilet flushing after defecation in the bathroom of room 1206, while all bathroom doors and kitchen range hoods and exhaust fans were closed. During the same period, all kitchen range hoods and bathroom doors of rooms 206, 806, 1406, 1606, and 1706 were opened (doors of a separate kitchen were also opened), and the toilet flushing was staggered for a few seconds on different floors. The differences in air velocity/pressure in exhaust fans and floor drains as well as the number of aerosol particles in the bathrooms of rooms 206, 806, 1406, 1606, and 1706 were measured every 10 min. PM_10_ aerosol filter membrane samples were also collected for 1 h with medium flow samplers in bathrooms. After a 1-h experiment, swab samples were smeared from toilet surfaces, exhaust fans, and floor drains (see Supplementary Table 2 for details).

Instruments and materials used in all experiments were medium flow aerosol samplers (100 L/min, equipped with a TSP-PM_10_ cutting head, Wuhan Tianhong Instrument Co., HY-100WS and Qingdao Laoying Environmental Technology Co., Ltd., type 2037), particle counters (particle size ranges are 0.3, 0.5, 1.0, 2.0, 3.0, and 5.0 μm; CLJ-H630, Honri Airclean Tech., China); a differential pressure meter (Testo 512, Germany); anemometer (QDF-6, Qingdao Juchuang Environmental Protection Group Co., Ltd., China); quartz filter membranes (diameter 90 mm, PALL Company, USA); and smeared swabs.

After these field experiments, a fluorescent microscope (OLYMPUS CX41) and a camera (OLYMPUS E330) were used to observe fluorescent polystyrene microspheres in different samples, such as smeared swabs collected from the floor drains of bathrooms and exhaust air ducts/exhaust fans as well as aerosol filters. All the observed samples were recorded by electronic photos.

### 2.2. Statistical analysis

WPS Office software and SPSS 26.0 were used for data entry, cleaning, and graphing. Descriptive epidemiological methods were used to analyze the historical epidemiology, environmental risks, and nucleic acid detection results of the two cluster outbreaks, and the possible viral aerosol transmission routes were also analyzed with simulated experimental data.

## 3. Results

### 3.1. Results in building A

In Scenario I of building A, exhaust fans in rooms 119 and 223 were damaged and were not installed in rooms 421 and 323; exhaust vents were blocked in rooms 521 and 621. During the experiment, there was no defecation or toilet flushing in any of the rooms. Researchers simulated the exhalation of simulants in the bathroom of room 323, and no fluorescent microspheres were observed on the swabs smeared from exhaust outlets in bathrooms of rooms 119, 223, 421, 521, and 621 (Table 1). There were no significant changes in air velocity, differential pressure measured at the exhaust outlets, or the number of particles monitored in the bathrooms. The results indicated that, after the infection occurred in building A, because there was no power drive in the exhaust air shaft and the exhaust fan in each room was missing or damaged, the air flow was relatively static and the aerosols produced by breathing were difficult to spread to different rooms in the same vertical apartment through the exhaust air shaft.

**Table 1:** The results of on-site simulation experiments in building A.

**Number of rooms**	**Results**	
	**Scenario I**	**Scenario II**
	**Smeared swab from bathroom exhaust duct**	**Aerosol filter membrane**
119	Not observed	Observed
223	Not observed	Observed
421	Exhaust air shaft was sealed	Observed
521	Not observed	Observed
621	Exhaust air shaft was sealed	Observed

Fluorescence microspheres were not observed in all background samples.

**Figure 2:**
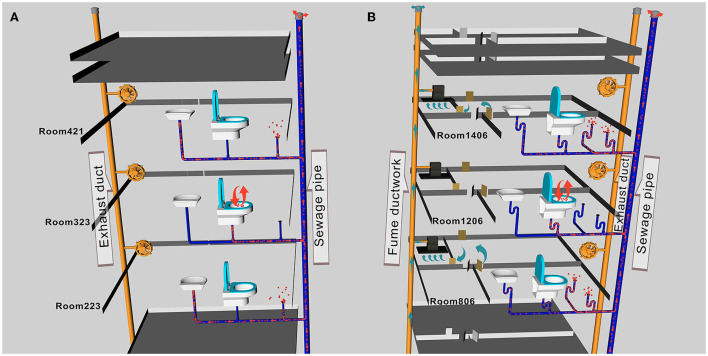
A schematic diagram of aerosol transport pathways in buildings **(A, B)**. **(A)** Viral aerosol simulants entered each bathroom through the path of “excretion of virus through feces-toilet flushing-sewage pipe-floor drain without a water seal.” **(B)** The path of viral aerosol simulant entered each room was through “excretion of virus through feces-toilet flushing-sewage pipe-floor drain without a water seal”.

**Figure 3:**
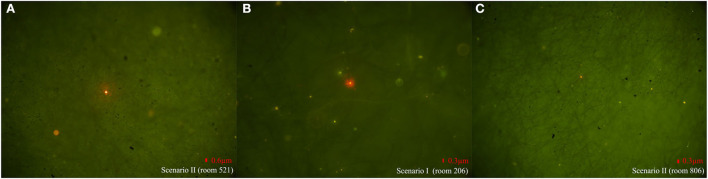
Representative photos of fluorescent microspheres tracked by different sampling methods in different rooms in two scenarios. **(A)** Fluorescent microspheres were observed on aerosol filter membrane samples in Scenario II of building A. **(B)** Fluorescent microspheres were observed on aerosol filter membrane samples in Scenario I of building B. **(C)** Fluorescent microspheres were observed on smear swab samples collected from the floor drain in Scenario II of building B.

In Scenario II of building A, researchers discharged fluorescent simulants by exhaling and flushing the toilet after defecation in the bathroom of room 323 and then flushing every 10 min. Within 30 s after every toilet flushing, the floor drain air velocity changed significantly in rooms 119, 223, 421, and 521 except for room 621 (room 223, for example, Supplementary Figure 1), where the pressure difference across the floor drain was positive, and the air velocity and pressure difference at the exhaust outlet did not change significantly, which suggested air flow entering the bathroom through the floor drain. The number of particles in the bathrooms on each floor changed significantly, and the concentration of small particle aerosols increased significantly (room 223, for example, Supplementary Figure 2). After a 1-h simulation experiment, fluorescent microspheres were observed in the aerosol filter membrane samples collected from the bathrooms of the same vertical apartments in building A (Table 1, **Figure 3A**), which revealed that simulants could enter these bathrooms through a sewage pipe. No fluorescent microspheres were observed in swabs smeared from an exhaust air shaft outlet, but some were observed in swabs smeared from the floor drain. It indicated that viral aerosol simulants entered each bathroom through a path of “excretion of virus through feces-toilet flushing-sewage pipe-floor drain without a water seal” (Figure 2A).

### 3.2. Results in building B

In Scenario I of building B, doors and windows in the room were closed, all kitchen and bathroom doors were opened, and the range hoods were turned on in all experiment rooms except room 1206. Researchers simulated exhaling viral simulants in the bathroom of room 1206 with the door closed. The results showed that there were no changes in the air velocity of an exhaust outlet and a floor drain in different time periods, and the changes in the number of particles were also not obvious. After a 1-h experiment, fluorescent microspheres were observed in swab samples smeared from the floor drains in rooms 206, 806, 1406, and 1706 (the floor drain in room 1606 was sealed and no samples were taken) and from the exhaust outlets in rooms 206, 806, and 1706. Fluorescent microspheres were observed in aerosol filter membrane samples collected from all bathrooms (Table 2, Figure 3B). It indicated that the negative pressure in the rooms caused by turning on range hoods could lead to viral aerosol simulants, which entered the bathrooms through a sewage pipe (11). Because the exhaust fans installed in the bathroom were equipped with two check valves that allowed only bathroom gas to drain out but not outside air to drain, viral aerosol simulants were unlikely to enter bathrooms through the exhaust fans. On the other hand, although the floor drain had a *U*-shaped trap, the floor drain in the dry zone was not used at ordinary times, and the *U*-shaped trap was dry, viral aerosol simulants were more likely to spread outside the bathroom through floor drains. The fluorescent microspheres observed on exhaust fan swabs were probably due to the adsorption of viral aerosol simulants on the surface of the exhaust fans.

**Table 2:** The results of on-site simulation experiments in building B.

**Number of rooms**	**Results**			
	**Scenario I**			**Scenario II**
	**Aerosol filter membrane**	**Smeared swab**		**Aerosol filter membrane**
		**Exhaust outlet**	**Floor drain**	
206	Observed	Observed	Observed	Observed
806	Observed	Observed	Observed	Observed
1406	Observed	Not observed	Not observed	Observed
1606	Observed	Not observed	Floor drain was sealed	Observed
1706	Observed	Observed	Observed	Observed

Fluorescence microspheres were not observed in all background samples.

In Scenario II of building B, the state of each room remained unchanged, and researchers discharged fluorescent simulants using exhalation and toilet flushing after defecation in the bathroom of room 1206. There were no obvious changes in the air velocity of an exhaust outlet at different flushing times, but the air velocity of a floor drain changed significantly (room 206, for example, Supplementary Figure 3). The number of particles changed as well (room 206, for example, Supplementary Figure 4). After a 1-h experiment, fluorescent microspheres were observed in the swab samples smeared from the floor drain in all bathrooms and were also observed in aerosol filter membrane samples collected from the bathrooms (see Table 2, Figure 3C to see some results from the swab samples and the aerosol filter membrane samples), which indicated that aerosol simulants could enter different rooms through a sewage pipe. Fluorescent microspheres were observed on all swabs smeared from floor drains, indicating that the path of viral aerosol simulants entering each room through “the excretion of virus through feces-toilet flushing-sewage pipe-floor drain without a water seal” (Figure 2B).

## 4. Discussion

### 4.1. Effects of bathroom exhaust air shafts on the spread of viral aerosols

It was reported that the spread of viral aerosols through exhaust air shafts could cause the cluster outbreak of COVID-19 (12). Through the results of an epidemiological investigation on close contacts and nucleic acid tests for the environment, it was inferred that the first case was possibly infected through aerosol particles in the ventilation duct system of a quarantined hotel. However, aerosol samples from the rooms of a quarantined hotel were not collected and evidence of direct aerosol transmission was lacking. In our study, viral aerosol simulants were used to compensate for the lack of aerosol samples. Building A is a six-story apartment building with only one bedroom and one bathroom, and there are several rooms on both sides of a long corridor. There are no windows at the two ends of the corridor, and the ventilation in the corridor is poor. Rooms 119, 223, 323, 421, 521, and 621 have the same layout on different floors (in this study we refer to them as vertically distributed). The windows in each room are double-glazed, and the seams are sealed with sealant. The bathroom has no window and only one door. There are sewage pipes and exhaust ducts in the bathroom, but the installed exhaust fans are either broken or blocked. The results revealed that, when the exhaust air shaft was not driven by power (no exhaust fan or the exhaust fan was not turned on), the indoor and outdoor pressures were balanced, the airflow in the air shaft was relatively static, and aerosols generated by breathing were difficult to spread through the exhaust air shaft. The ventilation rate and air flow mode were important factors in the transmission of viral aerosols to an indoor environment (13, 14).

The cluster outbreak occurred in unit six of building B. This unit has the same room layout with two bedrooms, a living room, a bathroom, and a kitchen, and the indoor area is about 78 m^2^. Each room has windows open to the outside, which have three layers of glass. Windows and door seams are sealed with sealing strips. The kitchen is equipped with a range hood, which is connected to the fume ductwork through a hose. There are three floor drains in the bathroom, and the floor drains are connected to *U*-shaped traps. The bathroom is equipped with an exhaust fan, which was connected to an exhaust duct through a hose. The existence of two check valves between the exhaust fan and the exhaust duct was to ensure that the airflow could only be discharged to the outside. An outlet of the fume ductwork, the sewage pipe, and the exhaust duct all were on the roof of building B. The indoor airtightness is good when all doors and windows in rooms were closed. Experimental results from building B showed that, when the doors and windows were closed and the range hoods were turned on, it could cause a slight negative pressure in the rooms. Although the air velocity from the exhaust outlet and floor drain, as well as the number of particles in bathrooms, did not change, fluorescent microspheres were observed on the aerosol filter membranes and on the smeared swab samples of some bathrooms of vertical-type apartments on different floors. It indicated that the negative pressure in the room could cause the viral aerosol simulants to spread into the bathroom through a sewage pipe. The outbreak of COVID-19 was reported in the vertical-type apartment of high-rise buildings (15, 16). In such buildings, the different vertical-type apartments were connected by the same air shaft, indicating a risk of aerosol transmission related to the exchange of exhaust air shafts (17). In our research, the exhaust fan installed in the bathroom was equipped with two check valves, which viral aerosols less likely to spread through the exhaust air shaft. On the other hand, although the floor drains had *U*-shaped traps, they did not have a water seal, making it more likely that the simulants spread through the floor drain. The fluorescent microspheres observed on smeared swabs from exhaust fans were probably due to the adsorption of viral aerosol simulants on the exhaust fan surface.

### 4.2. Influence of bathroom sewage pipes on the spread of viral aerosols

During experiments to simulate exhalation or defecation with toilet flushing (18), the pressure generated by flushing caused viruses in the feces to escape with aerosols (1, 19). In our study, fluorescent microspheres were observed in aerosol filter membrane samples collected from all bathrooms in the same vertical apartments, indicating that viral aerosol simulants could spread to the bathrooms through a sewage pipe. It was found that, after flushing, the air velocity of the floor drains changed in almost all the bathrooms on different floors. The pressure difference was positive, which revealed that there was airflow into the room from the floor drain. Floor drain air velocity changed more than that without flushing. Because the airflow strongly affected the transmission of aerosol simulants (20), the flushing behavior intensified the spread of aerosols from the floor drain to the bathroom. Fluorescent microspheres were observed in smeared swabs from the floor drain, which also proved that the transmission path of viral aerosol simulants spread to each room through “excretion of virus through feces-toilet flushing-sewage pipe-floor drain without a water seal” (Figure 2). The results were consistent with research in South Korea (2), that SARS-CoV-2 could spread vertically *via* aerosols between the same types of rooms on different floors.

### 4.3. Influence of indoor aerosol particle size distribution characteristics on the diffusion of viral aerosols

In our previous studies, the aerosol particle size spectrum was monitored in different places such as office buildings or hospital outpatient wards in Beijing and a high-rise residential building in Shenzhen, one of the southern cities of China. Compared with the average number proportion of 0.3, 0.5, 1.0, 2.5 (or 2 μm), and 5 μm across a total of five aerosol particles, the proportion of 0.3 μm was the highest (53.2% in an office building, 75.5% in a hospital outpatient hall, and 70.6% in a high-rise residential building), followed by 0.5 μm (41.2% in an office building, 20.6% in a hospital outpatient hall, and 25.7% in a high-rise residential building) and 1 μm (5.5% in an office building, 3.6% in a hospital outpatient hall, and 1.9% in a high-rise residential building). In this study, the proportion of numbers of 0.3, 0.5, and 1.0 μm monitored in a high-rise residential building in Changchun accounted for 57.7, 28.0, and 7.6%, respectively, showing the same distribution characteristics, as shown in previous studies. Previous monitoring results showed that, in different indoor places with different environmental factors or people's activities, aerosols with a smaller size were dominating and aerosol particles of 0.3 μm accounted for the largest proportion. Particle counters were used to measure the concentration of fluorescent microspheres in our experiments. Two integrated wideband bioaerosol sensors were utilized to measure indoor and outdoor particulate matter and fluorescent biological airborne particles (FBAPs), arranged in size from 0.5 to 20 μm, according to the size-resolved data, FBAPs dominated above 2 and 3.5 μm indoors and outdoors, respectively (21). Using the three-dimensional (3D) fluorescent microscopic passive testing method, Li et al. also proposed for the first time to quantify size-resolved splashed cooking oil droplets on the surfaces of a countertop, wall, and dummy considering the influence of the range hood (22). The technique can also be used to test fluorescent microspheres.

Previous studies showed that the infectivity of SARS-CoV-2 peaked 2 days before and persisted 1 day after symptoms appeared (23). In March 2022, we investigated the occurrence of COVID-19-infected cases on different floors of the same apartment type in a high-rise residential building in Shenzhen. In Changchun, the epidemiological characteristics of infected cases in a high-rise residential building were similar to those in Shenzhen, where all initial infected cases were transported 2 days after onset. Before being transported, infected cases could produce large amounts of viral aerosols in a bathroom by breathing, talking, coughing, sneezing, or using the toilet (24–26). It was experimentally confirmed that the half-life of SARS-CoV-2 absorbed in aerosols were ~1–3 h (27, 28). If viral aerosols from infected cases accumulated in poorly ventilated spaces, they could spread over short and long distances (such as sewage or exhaust air shafts) and still remain infectious. This was consistent with the fact that infected cases were found later in the same apartment type and on different floors of two high-rise residential buildings in both Shenzhen and Changchun.

Relevant studies on the distribution of viral aerosols confirmed that the viral genome was mostly detected in aerosols with a size < 5 μm and not in larger particle sizes (29). In particular, fine aerosol particles contained more viral ribonucleic acid (RNA) (30), and infectious viruses were recovered in aerosols ranging from 0.25 to >4 μm (31–33). Usually, as concentrations of atmospheric particulate matter decreased, the amount of indoor aerosols with different particle sizes also decreased. By analyzing the amount of aerosol particles in the bathrooms of high-rise residential buildings in Shenzhen and Changchun, it showed that the atmospheric concentrations of PM_10_ (0.038 mg/m^3^) and PM_2.5_ (0.03 mg/m^3^) during on-site monitoring in Changchun were higher than those in Shenzhen (0.016 and 0.009 mg/m^3^). The number of indoor aerosols with larger particle sizes (1.0 and 2.0 μm) also had the same trend, but the amount of small particles (0.3 and 0.5 μm) was significantly lower in Changchun than in Shenzhen. The results indicated that, in a relatively closed environment, the amounts of indoor aerosol particles were not only affected by outdoor particles but were also related to indoor factors such as building structure, types of doors and windows, ambient temperature, humidity, personnel activities, and so on. In northeast China, it was usually windy and cold weather in winter with a long heating period. The concentrations of outdoor particulate in northeast China were usually higher than those in southern China. Closing doors and windows for a long time in winter could decrease the concentration of indoor particles from outdoors, but the amount of aerosol particles produced by indoor activities was still at a high level.

According to previous literature studies, healthy adults inhale ~8, 22, and 33 L/min during light, moderate, and heavy physical activities, respectively (34), and different particle sizes and concentrations of inhalable aerosols are then produced. One study found that the SARS-CoV-2 inhale emission rate into the air was the highest, up to 105 viruses/min, during the early stages of COVID-19 (35). In another study (25), 36 volunteers aged 18–29 years with no evidence of previous infection or vaccination were inoculated with 10 TCID50 of a wild-type virus (SARS-CoV-2/human/GBR/484861/2020), participants became infected, with a pronounced increase in viral load (VL) and a peaking ~5 days after inoculation. However, the concentration of viral aerosols that infect humans has not been reported in the literature. When there were cases infected with COVID-19, with poor indoor ventilation and more than 1 h of exposure, the transmission of viral aerosol transmission through a small particle size (36) was more likely.

In view of the existence of “excretion of virus through feces-toilet flushing-sewage pipes-floor drain without a water seal” transmission paths in residential buildings, it is recommended that enterprises (apartment buildings) or community properties (residential buildings) should check for *U*-shaped traps and floor drain water seals in the drainage system in accordance with the water supply and drainage design drawings. If these measures are not available, they should be renovated as soon as possible. It is recommended to install *U*-shaped traps to ensure the sealing of floor drains in buildings where conditions permit, and to install deodorizing floor drains in buildings where reconstruction is difficult. In northeast China, houses have good airtightness to keep warmth in winter, and doors and windows are usually closed. When cooking and the range hoods are turned on for a long time, it will cause indoor negative pressure. Thus, viral aerosols in the sewage pipe could enter the bathroom through the floor drain without a water seal, causing the vertical transmission of viral aerosols on different floors (sharing a sewage pipe). It is recommended to keep the indoor windows open, especially the bathroom, and close the bathroom door when the range hood is turned on, and at the same time, ensure that the bathroom floor drains with a water seal. If there is no window that opens to the outside in the bathroom, it is necessary to turn on the exhaust fan for ventilation.

This study has several limitations. Because live viruses cannot be used in the field and fluorescent polystyrene microspheres cannot completely simulate live viruses and their loadings in aerosols, the risk of infection cannot be quantitatively predicted, and even quantification of fluorescent particles on the membrane cannot account for the amount of viral infection. In addition, the simulated aerosol volume differs from the actual situation of confirmed cases. Therefore, on-site simulation experiments can only provide a possible route of transmission and factors influencing viral aerosols.

## Data availability statement

The original contributions presented in the study are included in the article/Supplementary material, further inquiries can be directed to the corresponding authors.

## Author contributions

QW and DX proposed research hypotheses. QW, DX, and XL designed the present study. BS provided the surveillance data of the outbreak. XL, BS, KL, QW, and DX drafted the manuscript, drafted a field investigation proposal, and involved in the laboratory analysis of samples. XL, BS, QW, and DX organized the field implementation. XL, BS, KL, YZ, YS, CL, TS, XW, YH, QW, and DX conducted on-site simulation experiments and sample collection. XL, BS, KL, JC, QW, and DX analyzed the data, and drafted the figures and tables. All authors approved the submitted version and agreed to be personally accountable for their own contributions.
