# Supplementary material for: Research on the relationship between architectural features in northeast China and vertical aerosol transmission of COVID-19
Source: Front Public Health. 2023 Jan 12;10:1052610. doi: 10.3389/fpubh.2022.1052610 (PMC9881651; doi:10.3389/fpubh.2022.1052610)
Supplement: Supplementary file 1 [file Data_Sheet_1.docx]

**Supporting Information**

Table S1. The combined scheme of aerosol field simulation experiments in scenario II of the multistoried apartment building (A)

| Room No. | 119 | 223 | 323 | 421 | 521 | | 621 | |
| --- | --- | --- | --- | --- | --- | --- | --- | --- |
| 0min | Except for 323, the rooms on other floors are equipped with medium flow samplers. Measuring background values (temperature and humidity, particle count) before the experiment. After the simulation experiment started, according to the instructions, the researchers in each floor simulated toilet flushing, recorded the video of the air velocity/pressure difference of the floor drain and the exhaust air duct, and measured the number of particles. | | | | | | | |
| 10min | Flushing  +3s | Flushing  +9s | Respiration + Defecation,  Flushing 0s | Flushing  +6s | | Flushing  +12s | | / |
| 20min | Flushing  +12s | Flushing  +6s | Respiration + Defecation,  Flushing 0s | Flushing  +9s | | Flushing  +3s | | / |
| 30min | / | / | / | / | | / | | / |
| 40min | Flushing  +3s | Flushing  +9s | Respiration + Defecation,  Flushing 0s | Flushing  +6s | | Flushing  +12s | | / |
| 50min | Flushing  +12s | Flushing  +6s | Respiration + Defecation,  Flushing 0s | Flushing  +9s | | Flushing  +3s | | / |
| 60min | / | / | / | / | | / | | / |
| * Read and photograph the bathroom floor drain, air velocity/pressure difference, and particle numbers every 10 minutes.  * *Before flushing, place the anemometer probe at the air outlet, turn on the mobile phone in advance to shoot and record until 30s after flushing.  * *Flushing: Press the toilet flush button. | | | | | | | | |

Table S2. The combination scheme of aerosol field simulation experiments of scenario II in the high-rise building (B)

| Room No. | 206 | 806 | 1206 | 1406 | 1606 | 1706 |
| --- | --- | --- | --- | --- | --- | --- |
| 0min | Except for 1206, the rooms on other floors are equipped with medium flow samplers. Measuring background values (temperature and humidity, particle count) before the experiment. After the simulation experiment started, according to the instructions, the researchers in each floor simulated toilet flushing, recorded the video of the air velocity/pressure difference of the floor drain and the exhaust air duct, and measured the number of particles. | | | | | |
| 10min |  | Flushing  +3s | Respiration + Defecation,  Flushing 0s |  | Flushing  +6s |  |
| 20min | Flushing  +3s |  |  | Flushing  +6s |  |  |
| 30min |  | Flushing  +3s |  |  | Flushing  +6s | Flushing  +9s |
| 40min | Flushing  +9s |  |  | Flushing  +3s |  | Flushing  +6s |
| 50min |  | Flushing  +9s |  | Flushing  +3s | Flushing  +6s |  |
| 60min | Flushing  +3s | Flushing  +6s |  | Flushing  +9s | Flushing  +12s | Flushing  +15s |
| * Read and photograph the bathroom floor drain, air velocity/pressure difference, and particle number every 10 minutes.  * *Before flushing, place the anemometer probe at the air outlet, turn on the mobile phone in advance to shoot and record until 30s after flushing.  * *Flushing: Press the toilet flush button. | | | | | | |

Table S3. Positive results for environmental samples

| A | | | |
| --- | --- | --- | --- |
| Sample location | Sample type | O gene | N gene |
| 119 Bathroom toilet | swab samples | 37 | 37 |
| 119 Bathroom Sink | swab samples | 36 | 36 |
| 223 Bathroom Sink | swab samples | 33 | 33 |
| 323 Bathroom Sink | swab samples | / | 35 |
| 521 Bathroom shower handle | swab samples | 35 | 38 |
| 521 Bathroom exhaust port | swab samples | 35 | 35 |
| B | | | |
| 206 Environmental surfaces in bathroom | swab samples | 35 | 35 |
| 1406 Environmental surfaces in bathroom | swab samples | 37 | 37 |

|  |
| --- |

Figure S1. Scenario 2 of multistoried apartment building (A-room 223). The floor drain air velocity changes obviously (m/s).

|  |
| --- |

Figure S2. Scenario 2 of the multistoried apartment building (A-room 223). Variation trend of the number of aerosol particles with different particle sizes over time, the concentration of small-particle aerosols increases significantly.

|  |
| --- |

Figure S3. Scenario 2 of the high-rise building (B-room 206). The floor drain air velocity changes obviously (m/s).

|  |
| --- |

Figure S4. Scenario 2 of the high-rise building (B-room 206). Variation trend of the number of aerosol particles with different particle sizes over time, the concentrations of aerosols of all particle sizes increased significantly.
